# Motivation to comply with task rules and multitasking performance: The role of need for cognitive closure and goal importance

**DOI:** 10.1007/s11031-018-9678-2

**Published:** 2018-02-28

**Authors:** Ewa Szumowska, Małgorzata Kossowska, Arne Roets

**Affiliations:** 1 Philosophy Department, Institute of Psychology, Jagiellonian University, Ingardena Str. 6, 30-060 Kraków, Poland; 2 Department of Developmental, Personality, and Social Psychology, Ghent University, Ghent, Belgium

**Keywords:** Motivation to comply with task rules, Multitasking performance, Need for closure, Goal importance, Effort

## Abstract

In three studies, we examined the role task rules play in multitasking performance. We postulated that rules should be especially important for individuals highly motivated to have structure and clear answers, i.e., those high on need for cognitive closure (NFC). High NFC should thus be related to greater compliance with task rules. Specifically, given high goal importance, NFC should be more strongly related to a multitasking strategy when multitasking is imposed by the rules, and to a mono-tasking strategy when monotasking is imposed by the rules. This should translate into better multitasking or mono-tasking performance, depending on condition. Overall, the results were supportive as NFC was related to a more mono-tasking strategy in the mono-tasking condition (Studies 1 and 2 only) and more dual-tasking strategy in the dual-tasking condition (Studies 1–3). This translated into respective differences in performance. The effects were significant only when goal importance was high (Study 1) and held when cognitive ability was controlled for (Study 2).

## Introduction

Multitasking is ubiquitous and today, more than ever, individuals as well as groups and organizations must attend to multiple tasks simultaneously (Bühner et al. 2006; Hambrick et al. 2010; Waller 2007). Therefore, more and more studies are conducted to identify those who are more likely to engage in multitasking (e.g. Sanbonmatsu et al. 2013) and those who are good at it (e.g. Bühner et al. 2006; Hambrick et al. 2010). However, whether people engage in multitasking and how they perform depends not only on one’s ability or motivation, but also on contextual factors. The role of the latter has not gained much attention in the research on predictors of multitasking performance. This issue has been recently raised in the literature on polychronicity, or the preference for multitasking (see König and Waller 2010, for overview). For years this variable has been treated as equivalent to multitasking behavior (e.g. Hall 1959) but recently researchers have argued that polychronicity does not always lead to multitasking and, even if it does motivate multitasking, it does not always lead to effective performance (König and Waller 2010; Waller 2007). Whether that is the case, depends on contextual factors such as environmental pressures to do several things at once. So, there might be situations in which people who enjoy multitasking do not engage in it or exhibit poorer multitasking performance. Likewise, there might be situations in which people otherwise not inclined towards multitasking engage in it and might even outperform those who multitask a lot (e.g. Ophir et al. 2009). These contextual factors might be especially important in case of individuals highly motivated to adhere to situational norms and rules, i.e. those with high levels of need for cognitive closure (NFC, Kruglanski 1990).

NFC is a basic motivational tendency to avoid and quickly reduce uncertainty. Previous studies have shown that NFC is related to greater adherence to situational norms, rules and compliance with tasks demands (e.g., Chiu et al. 2000; Fu et al. 2007; Jaśko et al. 2015; Jia et al. 2014; Kruglanski and Webster 1996) as well as a greater focus on the main task goal (Szumowska and Kossowska 2017). Sticking to a reliable norm or rule reduces uncertainty, which is the main goal of high NFC individuals (Kruglanski 2004). Therefore, high (compared to low) NFC individuals should be more motivated to comply with the task rules. This should translate into a more diligent adoption of the required task strategy and as a result, a better multitasking performance when multitasking is required and better monotasking performance when monotasking is required. However, in order to be motivated to attain a goal, one needs to find that goal important (Atkinson and Birch 1970; Brehm and Self 1989; Kruglanski et al. 2012). So, the hypothesized effects of NFC on multitasking performance should appear only when the task goal is important. When the task goal is not important, no effects of NFC should be found. Also, since we postulate differences in motivation, the effect of NFC should hold when cognitive ability is controlled for.

The present research adds significantly to the understanding of the relationship between NFC and multitasking performance by including contextual moderators such as task rules. It also emphasizes the role of the latter by showing that individuals who are otherwise not inclined toward multitasking (Szumowska and Kossowska 2016; Szumowska et al. 2017), might exhibit better multitasking performance under some circumstances. The results of the studies might also have implications for designing job environments in order to maximize performance of high NFC individuals in the multitasking context.

## Need for closure and motivation to adhere to task rules

NFC is a basic motivational tendency to avoid and reduce uncertainty, and has been typically related to rigidity in information processing (Kruglanski 1990; Kruglanski and Webster 1996; see also; Roets et al. 2015). As such it has been shown to influence many areas of a person’s functioning, e.g. decision making (Jaśko et al. 2015), creativity (Gocłowska et al. 2014), hypothesis generation (Mayseless and Kruglanski 1987), social beliefs (e.g., Kossowska and Van Hiel 2003), and group behavior (Kruglanski et al. 2006; see; Roets et al. 2015, for overview). Individual differences in NFC reflect dispositional variability in preference for order, predictability, tolerance of ambiguity, and closed-mindedness (Kruglanski 2004). People low on NFC are generally open to prolonging uncertainty, engage in more deliberative decision-making and flexibility of thought. By contrast, people high on NFC prefer order, predictability and quick decision-making, and they usually exhibit rigidity of thought and a greater preference for conformity (Kruglanski 2004).

A large body of research has also shown that high NFC individuals adhere to situational norms and rules to a greater extent than their low NFC counterparts. For example, Jia et al. (2014) showed that high NFC individuals readily complied with the experimenter in a lab setting, which had been demonstrated as a powerful norm (Milgram 1974), and their behavioral intention was guided by the normative information in the prisoner’s dilemma to a greater extent than that of low NFC participants (Jia et al. 2014, Studies 1 and 2, respectively). The role of norms was also demonstrated in intercultural studies as high NFC participants were found to be more likely to exhibit attribution biases characteristic of the culture (Chiu et al. 2000) and conform to cultural norms in respect to conflict judgements (Fu et al. 2007). Also studies on group-centrism have revealed that individuals high on NFC strive for consensus which provides a stable, closure affording, shared reality that is pervasive and readily accessible (e.g., Kruglanski et al. 2006).

In a similar vein, Jaśko et al. (2015) showed that individuals high on NFC behaved in line with an accessible and reliable rule in a decision-making task, even if the rule led to a more effortful strategy. In fact, individuals high on NFC exhibited a longer decision-making process and extended information search in a condition in which this was a rule. The opposite effect took place in a condition in which the rule advised limited information search. These differences were not significant for individuals low on NFC, suggesting that these participants are less dependent on situational rules than their high NFC counterparts.

The above studies show that NFC is related to greater adherence to norms and rules, even if that requires more effort. Sticking to a reliable rule reduces uncertainty, the main epistemic goal of high NFC individuals (Kruglanski 2004). The striving of high NFC individuals to reduce uncertainty has been argued in many studies (Czernatowicz-Kukuczka et al. 2014; Jaśko et al. 2015) and biological underpinnings of this tendency have been postulated (Kossowska et al. 2014). So, it is because of the uncertainty reducing potential of norms and rules that high (but not low) NFC individuals are motivated to adhere to them.

Based on these insights, we expect that when presented with multiple tasks, high NFC individuals will be more motivated to rely on accessible rules, or explicit task demands, and behave in line with them. That is, they should exhibit better multitasking performance when a task requires the same, and better monotasking (worse multitasking) performance when focusing on one task is required. We thus assume that the NFC related differences in performance would stem from a greater motivation to comply with the task rules, i.e., compliance with the task rules should mediate the relationship between NFC and performance.

## The role of goal importance

Individuals are motivated to invest effort in attaining a goal only when they find that goal important (Brehm and Self 1989). According to traditional motivational theories (e.g., Atkinson and Birch 1970; Lewin et al. 1944; Vroom 1964) one of the most important determinants of motivation to attain a given goal is its perceived value, or importance. More recently, the Cognitive Energetics Theory (Kruglanski et al. 2012) similarly assumes that goal importance is one of the factors driving cognitive effort (as opposed to factors restraining it). The more important a certain goal is to a person, the more likely they are to invest effort to attain it. Also, according to the Motivational Intensity Theory (Brehm and Self 1989; Wright and Brehm 1984), importance of a given goal determines the level of potential motivation, that is the maximal amount of effort one is willing to invest in a given activity. The higher goal importance, the greater the motivation to attain this goal, and thus willingness to invest effort (when effort in required). Therefore, we can expect that high NFC individuals will be motivated to invest effort in order to comply with the task rules to attain the task goal—but only when they find the task goal important.

In support of this, a study by Viola et al. (2015) showed that high (as compared to low) NFC individuals invested more effort in the random dot motion task when the outcome relevance was high in comparison to when the outcome relevance was low. Also, a study by Sankaran et al. (2017) showed that perceived importance of the task goal moderated the relationship between NFC and effort invested in the task. Specifically, high NFC was related to lower effort invested in the task but only when the task goal importance was low. When the task goal importance was high, no effort reducing tendency of high NFC appeared (Study 1). Moreover, high NFC individuals invested more effort when only by doing so could they attain the task goal (Study 2).

In light of the above, we expect that the hypothesized relationship between NFC and multitasking performance mediated by compliance with task rules will be moderated by goal importance in a way that significant effects should be found only when the task goal is highly important to a person. By contrast, when the task goal is of little importance, there should be no significant effects of NFC on multitasking performance. High NFC individuals should thus exhibit a better multitasking performance only when the task goal is important and requires multitasking. When the task goal is important but monotasking is required, high NFC individuals should exhibit better monotasking performance. No effects of NFC should be found when the task goal is of little importance.

## Overview of the studies

In order to test our hypotheses, we presented participants with a dual-task paradigm in which two tasks were displayed simultaneously. In Study 1, we manipulated both task rules and goal importance. We manipulated task rules by instructing participants to perform both presented tasks as best as possible (dual-tasking condition), or to focus only on one of them (mono-tasking condition). We manipulated goal importance by making the task more or less relevant to a person, i.e., we told participants that the task measured a very important ability predictive of future outcomes (high goal importance condition) or that it was a pilot test of a filler task for future studies (low goal importance condition). On task completion, participants were asked about strategy they used (self-reported focus on one task vs. performing the two), which served as a measure of compliance with task rules.

We hypothesized that NFC would be related to a greater compliance with task rules, i.e. it would be related to focusing more on one task in the mono-tasking condition and performing both tasks at the same time in the dual-tasking condition, but only when the task goal was important. We also predicted that differences in strategy would translate into differences in performance, i.e. NFC should be related to better multitasking when multitasking is required and to better performance on the target task when focusing on one task is required. Again, effects should be present only in high (but not low) goal importance condition.

In Study 2, we wanted to replicate the results of Study 1 pertaining to the hypothesis that higher NFC is related to a greater motivation to adhere to task rules in the high goal importance condition. All participants were told that the task measured an important ability predictive of future outcomes. Additionally, we controlled for participants’ working memory capacity, a best ability predictor of multitasking performance (Bühner et al. 2006; Colom et al. 2010; Hambrick et al. 2010; König et al. 2005). By doing so, we wanted to test whether the effects of NFC held when participants’ cognitive ability was controlled for. In Study 3, we again manipulated task rules and asked participants to either mono- or dual-task. However, this time we used a dual-task consisting of two tasks of equal difficulty, so that dual-tasking reflected equal distribution of attentional resources between the two tasks rather than distributing resources according to task difficulty (which was the case in previous studies).

### Study 1

The aim of this study was to test whether compliance with task rules mediated the relationship between NFC and multitasking performance depending on task rules and goal importance. There were four conditions: 2 (mono-tasking or dual-tasking as a rule) × 2 (low vs. high goal importance). We expected that NFC would be related to greater compliance with task rules, and hence better multitasking performance in the dual-tasking condition and better performance on a single task in the mono-tasking condition, but only when goal importance was high. When goal importance is lower, people are not willing to invest effort. Therefore, we expected no effects of NFC in this condition.

#### Participants

Two hundred and six^1^ adults (160 women, 46 men) aged between 18 and 63 (*M* = 23.33, *SD* = 4.86) took part in the study. They were recruited by an announcement and given a monetary compensation of 2.5 EUR in exchange for participation in the study. The study was conducted in line with the local Faculty Ethics Committee with written informed consent from all subjects.

Since two participants had a very high number of wrong answers in in the verification task (> 100, vs. *M* = 8.46 for the rest of the sample, task description in the “Measures” section), they were deleted from further analyses. Also, the oldest participant was excluded, as (s)he considerably differed in this respect from the rest of the sample. Thus, the final sample comprised *N* = 203 subjects (158 women, 45 men) aged between 18 and 47 with the mean of *M* = 23.12 (*SD* = 4.01).

#### Measures

##### Need for closure

To measure NFC, we used the Need for Cognitive Closure Scale (Webster and Kruglanski 1994). The scale consists of five subscales: (1) preference for order and structure in the environment, (2) predictability of future contexts, (3) affective discomfort occasioned by ambiguity, (4) closed-mindedness, and (5) decisiveness of judgments and choices. Since the decisiveness (e.g., Roets and van Hiel 2007) and closed-mindedness subscales (Neuberg and Newsom 1993) have been shown to measure not only motivational but also other aspects, we have included only first three subscales in our analysis (see also Kossowska et al. 2002; Neuberg et al. 1997; Roets et al. 2006, 2015, for more support of this selection). Sample items are: *I find that establishing a consistent routine enables me to enjoy life more* or *I don’t like to be with people who are capable of unexpected actions*. Answers are given on a 6-point Likert scale (from *definitely disagree* to *definitely agree*). A global score of need for closure was calculated by averaging answers to all items. The scale proved satisfactory reliability (Cronbach’s α = 0.87; *M* = 4.09, *SD* = 0.67). Higher scores indicate higher NFC.

##### Multitasking performance

**Fig. 1:**
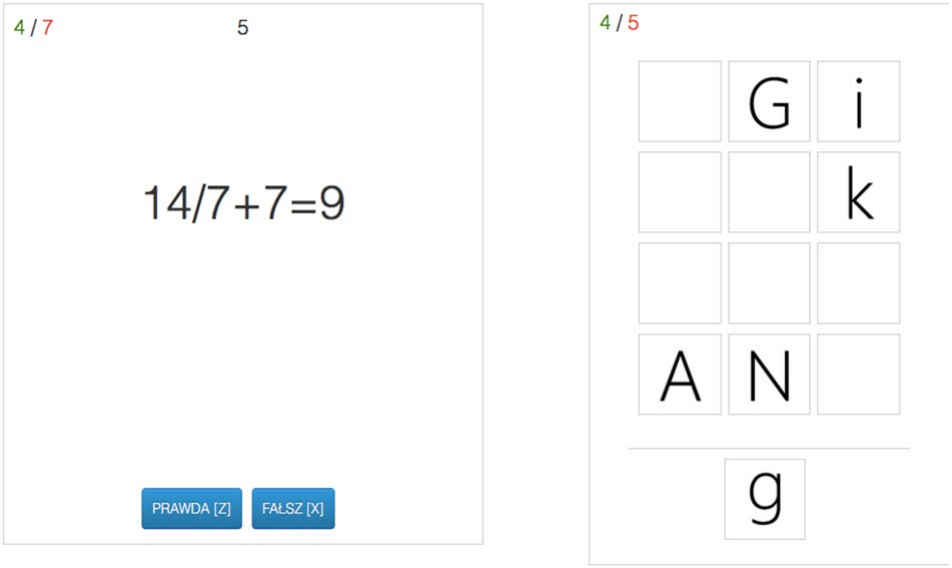
Dual-task procedure used in Studies 1 and 2. On the left, a verification task is presented. One needs to verify a mathematical expression by pressing either a “Z” (for true) or “X” (for false) key on the keyboard. On the right, a searching task is presented. One needs to react to a letter identical with the probe (presented below the line) by pressing the space bar. The number of correct and incorrect answers is presented for each task separately (in the left corner of each task frame)

To measure multitasking performance, we used a dual-task consisting of a searching task and a verification task (a modified version of a task used in Szumowska and Kossowska 2017, and; Szumowska et al. 2017). The two tasks were presented on a computer screen at the same time, the searching task on the right and the verification task on the left (see Fig. 1). In the searching task letters were presented every 750 ms. Participants’ task was to react (by pressing a space button) to a letter identical with the probe displayed below the presentation grid. Probe letters changed every 20 s, for each probe there were 2 target and 18 non-target letters. There were maximally 5 letters presented in the grid at the same time. There were 15 probes in total.

On the left side of the screen mathematical expressions were presented one at a time. Participants needed to verify whether the expression presented was true or false. Each mathematical expression consisted of two operations (a combination of addition and subtraction or multiplication/ division and addition/subtraction). Answers were given by pressing the “Z” button for true and the “X” button for false. Expressions changed every 7.5 s or once a person had verified an expression.

Accuracies were recorded for each of the two tasks separately. Correct and incorrect responses were signaled (background of a given task subtly highlighted in green for correct or in red for incorrect responses).

##### Self-reported strategy

After performing the task(s), participants were presented with two items referring to the strategy they have used during task execution: *Which task did you treat as more important?* and *Which task did you focus on more?* Answers were given on a 10-point scale in which 0 stood for *I treated both tasks as equally important*/ *I focused on both tasks to the same extent*, whereas − 5 and 5 corresponded to giving priority to the searching or verification task, respectively. Responses to both items were averaged (the items were highly correlated, *r* = .80, *p* < .001) to obtain a score reflecting strategy adopted in the task. Greater negative scores indicate greater inclination towards the searching task and greater positive scores towards the verification task; zero indicates equal distribution of focus between the two tasks. The score was used as an indirect measure of compliance with the task rules.

The decision to use such a measure of strategy was based on the results of a pilot study in which we asked a different sample of participants (*N* = 89) to perform the same dual-task as the one used in this study in the dual-tasking condition (we informed participants that both tasks were equally important and both needed to be performed as best as possible). Upon task completion, we asked participants about the strategy they had used while performing the dual-task. Specifically, we asked them the following questions: (1) *Which task did you treat as more important?* (2) *Which task did you focus on more?* (3) *Which task was more difficult to you?* and (4) *Which task required more of your attention?* (all anchored − 5 for the verification and 5 for the searching task, respectively). Participants were also asked to indicate to what extent they agreed with the following statements: (1) *Although both tasks were equally important, to obtain best overall score one should focus more on the verification task;* (2) *Although both tasks were equally important, to obtain best overall score one should focus more on the searching task;* (3) *Because both tasks were equally important, to obtain best overall score one should focus on both tasks to the same extent;* (4) *In such tasks, the most important is to know what to focus on;* (5) *In such tasks, the most important is to equally divide attention between both presented tasks* (all anchored 1—*definitely disagree* to 7—*definitely agree*) and (6) *In my opinion, overall performance in this dual-task depends on*: performance in the searching task only (− 5), equally on performance in both tasks (0) and performance in the verification task only (5).

The results showed that participants treated the verification task as more important (*M* = 1.20, which was significantly different from 0 indicating equal importance of both tasks, *t* (88) = 4.90, *p* < .001). They also focused on the verification task to a greater extent than on the searching task (*M* = 1.70, which was significantly different from 0 indicating equal focus on both tasks *t* (88) = 6.48, *p* < .001). Participants also reported that the verification task was more difficult (*M* = 2.37, which was significantly different from 0 indicating equal difficulty of both tasks, *t* (88) = 8.26, *p* < .001) and required more of their attention (*M* = 2.78, significantly different from 0 indicating equal attentional demands of both tasks, *t* (88) = 10.56, *p* < .001). This suggests that the best strategy to efficiently perform this particular dual-task is to divide attention between the tasks according to their demands, i.e., focus slightly more on the verification task.

This is further supported by the observation that participants agreed more to the statement: *Although both tasks were equally important, to obtain best overall score one should focus more on the verification task* (*M *= 4.60) than to the statement: *Although both tasks were equally important, to obtain best overall score one should focus more on the searching task* (*M* = 2.97) or *Because both tasks were equally important, to obtain best overall score one should focus on both tasks to the same extent* (*M* = 3.87)—as indicated by the repeated measure ANOVA results: *F* (2, 87) = 30.72, *p* < .001; respective pairwise comparisons significant at *p* < .001 and *p* = .02. Participants also reported that, in their opinion, the overall score in the dual-task depended more on the verification task (*M* = 1.24, which was significantly greater from 0 indicating equal perceived influence of both tasks, *t *(88) = 6.73, *p* < .001). They also agreed more with the statement that in such tasks the most important thing was to know what to focus on rather than to equally divide attention between both tasks (*M* = 4.85 vs. *M* = 4.18, *F* (1, 88) = 6.90, *p* = .01).

Based on these findings from the pilot study, we decided to choose the searching task as the target task in the mono-tasking condition (as by default participants tend to focus more on the verification task and we wanted to capture a deliberate strategy implementation). We also assumed that in this particular task situation, mono-tasking strategy would be best reflected in focusing mainly on the searching task, whereas dual-tasking strategy would be best reflected by adjusting focus to the task demands, that is, focusing more on the verification task (especially that in the mono-tasking condition verification task was to be neglected).

#### Design and procedure

Before coming to the laboratory, participants were asked to fill in an online survey (Need for Cognitive Closure Scale and demographic variables). In the laboratory, they were asked to complete a dual-task procedure in one of the four conditions: 2 (mono-tasking vs. dual-tasking task rules) × 2 (high vs. low goal importance). We manipulated task rules by asking half of the participants to perform both presented tasks as best as possible (dual-tasking condition) or to focus only on the searching task (mono-tasking condition). To provide a cover story for such a manipulation, participants in the dual-tasking condition were told that the task measured the ability to perform *two* tasks at the same time, whereas participants in the mono-tasking condition were told that the task measured the ability to perform *one* task while it was presented along with another task. Therefore, participants in the dual-tasking condition were directly instructed to perform both tasks as best as they possibly could because the two tasks were equally important and contributed to the general score to the same extent, and participants in the mono-tasking condition were instructed to focus on the searching task because only this task contributed to the general score obtained in the study. However, they could also perform the verification task if they wanted to.

We manipulated goal importance by telling half of the participants that the task they were presented with measured an important and useful ability which, according to psychological research, is predictive of future academic and job outcomes (high goal importance). We told the other half of the participants that the task would be a filler procedure in future studies and the researchers just wanted to pretest it and check whether it was not too tiring (low goal importance). System randomly assigned participants to one of the four conditions.

After performing the task, participants answered two strategy items. They were also asked to rate to what extent the task was tiring, for consistency with the cover story. Then, they were debriefed and thanked. All computer tasks were displayed on 19 inches LCD monitors. The dual-task was programmed in JavaScript.

#### Results and discussion

To test our hypotheses, we regressed the strategy score on NFC, task rules (mono-tasking condition coded as 0, dual-tasking condition as 1), goal importance (low goal importance condition coded as 0, high goal importance condition as 1), and the interaction terms for all three variables. We expected that strategy used in the task would be significantly predicted by the three-way interaction in a way that high NFC would lead to more priority given to the searching task (i.e., negative relationship between NFC and the strategy score) in the mono-tasking condition and more distributed attention between the two tasks (i.e. positive relationship between NFC and the strategy score) in the dual-tasking condition, but only when goal importance was high. We did not expect significant effects of NFC on strategy in the low goal importance condition.

**Fig. 2:**
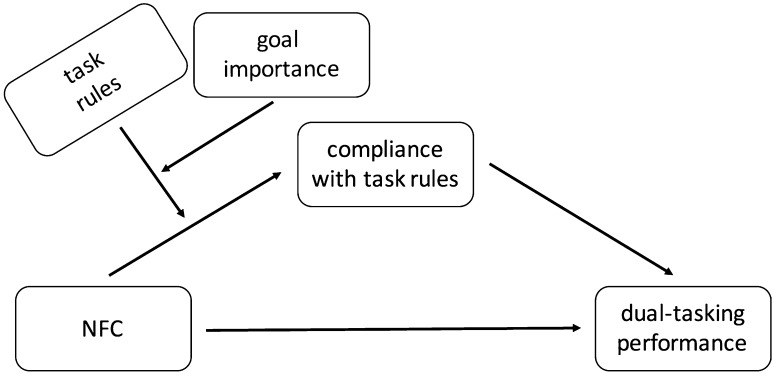
Theoretical model tested in Study 1

Next, we checked whether the strategy score mediated the relationship between NFC and performance conditionally on both task rules and goal importance. We thus tested a moderated mediation model as graphically presented in Fig. 2. We ran separate analyses for mono-tasking and dual-tasking performance. Accuracy on the searching task served as a measure of mono-tasking performance, as this task was the target one in the mono-tasking condition. To measure dual-tasking performance, we averaged percentage of correct answers in both tasks, as it was performance on both tasks that mattered for efficient dual-tasking. Additionally, we also report results for performance on each task separately in this condition.

Moderated mediation analyses were run with the use of the Process macro for SPSS version 2.13, model 11 (Hayes 2013). In all analyses 10,000 bootstrap samples for bias corrected bootstrap confidence intervals were used. All continuous variables were standardized before the analyses.

#### Effects on strategy

As presented in Table 1, a regression model for strategy yielded a significant three-way interaction. The two-way NFC × task rules interaction was close to significance, NFC × goal importance interaction was significant, and task rule × goal importance interaction was non-significant. There was also a significant main effect of task rules on strategy; the effects of NFC and task relevance were non-significant (see Table 1).

**Table 1:** Standardized regression coefficient for the model predicting strategy in Study 1 (*N* = 203)

	*β*	SE	*t*	*p*
Step 1^a^				
NFC	0.03	0.06	0.57	.569
Task rules	1.05	0.12	8.76	< .001
Task relevance	− 0.15	0.12	− 1.24	.216
Step 2^a^				
NFC	− 0.01	0.10	− 0.12	.901
Task rules	1.09	0.17	6.26	< .001
Task relevance	− 0.11	0.17	− 0.64	.524
NFC × task rules	0.10	0.12	0.77	.440
NFC × task relevance	− 0.01	0.12	− 0.07	.942
Task rules × task relevance	− 0.07	0.24	− 0.30	.762
Step 3^b^				
NFC	0.17	0.12	1.51	.133
Task rules	1.06	0.17	6.22	< .001
Task relevance	− 0.07	0.17	− 0.44	.066
NFC × task rules	− 0.29	0.16	− 1.74	.084
NFC × task relevance	− 0.45	0.18	− 2.55	.012
Task rules × task relevance	− 0.08	0.24	− 0.34	.731
NFC × task rules × task relevance	0.81	0.24	3.38	.001

^a^R^2^ = .29

^b^R^2^ = .33, ∆R^2^ = 0.04, *F* (1, 195) = 11.41, *p* < .001

**Fig. 3:**
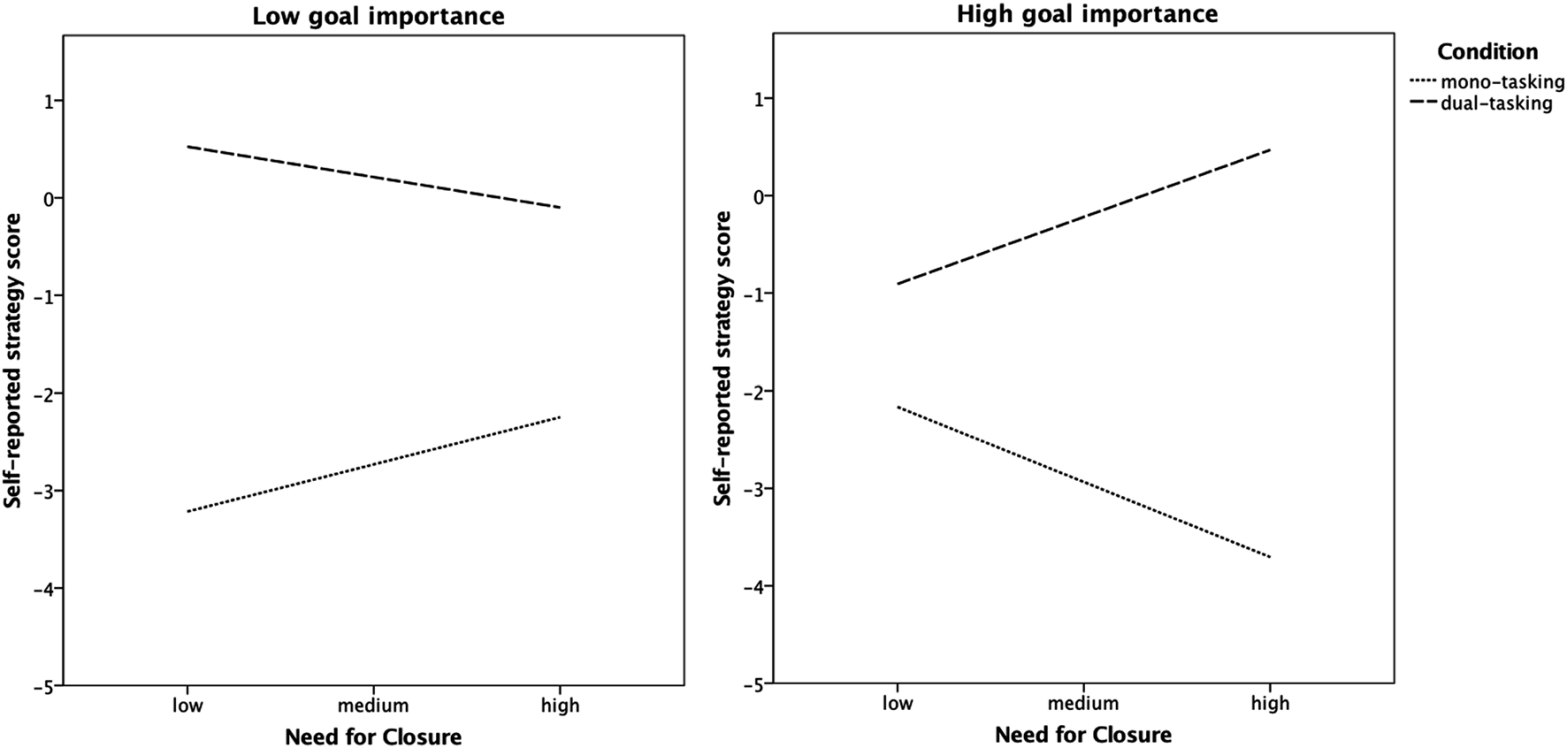
Relationship between need for closure and priority given to the searching versus verification task (measure of compliance with task rules) in both, mono-tasking and dual-tasking, conditions depending on goal importance (Study 1)

As for the three-way interaction, it indicates that, in line with our predictions, there is a negative effect of NFC on dual-tasking strategy score in the mono-tasking high goal importance condition, *β* = − 0.28, SE = 0.13, *t* = − 2.06, *p* = .04, 95% CI [− 0.54, − 0.01]. Thus, the higher NFC, the more focus on the searching task in the mono-tasking condition. The opposite effect emerged in the dual-tasking high goal importance condition, *β* = 0.25, SE = 0.11, *t* = 2.22, *p* = .03, 95% CI [0.03, 0.47]. So, the higher NFC, the more dual-tasking strategy in this condition (more focus towards the verification task). Both these effects suggest that NFC is positively related to compliance with task demands but only in the high goal importance condition. Both effects were non-significant in the low goal importance condition, *β* = 0.17, SE = 0.11, *t* = 1.51, *p* = .13, 95% CI [− 0.05, 0.40] for mono-tasking, and *β* = − 0.11, SE = 0.12, *t* = − 0.95, *p* = .34, 95% CI [− 0.34, 0.12] for dual-tasking conditions when goal importance was low. The interaction is graphically presented in Fig. 3. Additional inspection of differences between conditions showed that the differences between conditions were significant for low (*p* < .001) and high (*p* = .002) NFC individuals in the low goal importance condition and for high NFC individuals in the high goal importance condition (*p* < .001). The difference was not significant for low NFC individuals in the high goal importance condition (*p* = .07; for means see Fig. 3).

#### Moderated mediation effects on performance

Next, we tested moderated mediation models as presented in Fig. 2. We first analyzed mono-tasking performance, that is, the number of correct responses in the searching task. The analyses showed that the strategy significantly predicted mono-tasking performance, *β* = − 0.28, SE = 0.07, *t* = − 4.19, *p* < .001, 95% CI [− 0.42, − 0.15]. There was also a hypothesized indirect effect of NFC on mono-tasking performance mediated by strategy used in the task. It was positive in the mono-tasking high goal importance condition, *β* = 0.08, SE = 0.04, 95% CI [0.01, 0.18] and negative in the dual-tasking high goal importance condition, *β* = − 0.07, SE = 0.04, 95% CI [− 0.17, − 0.02]. No significant effects were found in both low goal importance conditions, *β* = − 0.05, SE = 0.04, 95% CI [− 0.15, 0.03] and *β* = 0.03, SE = 0.04, 95% CI [− 0.03, 0.12] for mono- and dual-tasking conditions, respectively. This suggests that the higher NFC, the better performance on the searching task in the mono-tasking condition and worse in the dual-tasking condition but only when goal importance is high. The direct effect of NFC was non-significant, *β* = − 0.09, SE = 0.07, *t* = − 1.38, *p* = .17, 95% CI [− 0.23, 0.04].

As for dual-tasking performance (averaged performance on both tasks), strategy was also a significant predictor, *β* = 0.18, SE = 0.07, *t* = 2.66, *p* = .008, 95% CI [0.05, 0.32]. Like in case of mono-tasking performance, no significant indirect effects of NFC were found when goal importance was low, *β* = 0.03, SE = 0.03, 95% CI [− 0.01, 0.11] and *β* = − 0.02, SE = 0.02, 95% CI [− 0.08, 0.02] for mono- and dual-tasking conditions, respectively. When goal importance was high, NFC was related to poorer dual-tasking performance in the mono-tasking condition, *β* = − 0.05, SE = 0.03, 95% CI [− 0.14, − 0.01]. On the contrary, when participants were asked to perform both tasks, high NFC was related to better dual-tasking performance, *β* = 0.05, SE = 0.03, 95% CI [0.01, 0.12]. These effects are indirect effects mediated by compliance with task rules. The direct effect of NFC was also significant, *β* = − 0.15, SE = 0.07, *t* = − 2.24, *p* = .03, 95% CI [− 0.29, − 0.02].

Similar results were found when performance on the verification task only was analyzed. Strategy was a significant predictor, *β* = 0.47, SE = 0.06, *t* = 7.70, *p* < .001, 95% CI [0.35, 0.60]. No significant indirect effects were found when goal importance was low, *β* = 0.08, SE = 0.07, 95% CI [− 0.05, 0.22] and *β* = − 0.05, SE = 0.06, 95% CI [− 0.17, 0.06] for mono- and dual-tasking conditions, respectively. When goal importance was high, NFC was related to poorer performance on the verification task in the mono-tasking condition, *β* = − 0.13, SE = 0.06, 95% CI [− 0.26, − 0.01] and better performance on this task in the dual-tasking condition, *β* = 0.12, SE = 0.05, 95% CI [0.03, 0.23]. The direct effect of NFC was equal to *β* = − 0.12, SE = 0.06, *t* = − 1.96, *p* = .05, 95% CI [− 0.24, 0.001]. The results thus suggest that high NFC participants ignored the verification task to a greater extent than their low NFC counterparts when they were instructed to do so (in the mono-tasking condition) and when following instructions was important to them (high goal importance condition). Therefore, they performed worse on this task in this situation. However, when they were asked to perform both tasks in parallel, they performed better on the verification task (dual-tasking high goal importance condition), which contributed to a better overall multitasking score. This better performance on the verification task in the dual-tasking condition was accompanied by poorer performance on the searching task, which suggests that performing one task along with another comes at the expanse of performance on this task. This is in line with numerous studies demonstrating the costs of performing two tasks at the same time (e.g., Kahneman 1973; Meyer and Kieras 1999; Navon and Gopher 1979; Navon and Miller 2002; Levy and Pashler 2008; Pashler 1984, 1994; Pashler and Johnston 1989; Tombu and Jolicoeur 2003). We should note, however, that the decrease in performance in the searching task was not dramatic (average performance on both tasks—due to their greater rule adherence—was still better for high NFC participants).

Overall, the results of the study indicate that NFC is related to greater compliance with task rules (greater focus on the target task in the mono-tasking condition and more dual-tasking strategy in the dual-tasking condition), but only when goal importance is high. When goal importance is low, no effects of NFC were found. Furthermore, high NFC individual’s greater compliance with the task rules in high goal importance condition translated into better performance, either mono-tasking or multitasking, depending on a condition. No differences in performance were found when the task goal importance was low.

Importantly, we should note that in this study we assumed that efficient multitasking denotes effective (but not necessarily equal) distribution of attentional resources between the tasks at hand. This assumption was based on the literature showing that how resources are distributed in a dual-task scenario depends on the difficulty of individual tasks and the cognitive engagement required in each of them (e.g., Courage et al. 2015; Kahneman 1973; Kemker et al. 2009; Luximon and Goonetilleke 2012; Navon and Gopher 1979; Navon and Miller 2002; Pashler 1984, 1994; Telford 1931; Tombu and Jolicoeur 2002, 2003). This was also supported by the results of the pilot study which showed that, in order to obtain best overall performance in the dual-task, one needed to focus slightly more on the verification task, as this task was more difficult. This was reflected in the strategy score we adopted (greater scores indicate more attention to the more difficult task). One should note, however, that although true for the dual-task we used, it is not necessarily the case in other dual-task situations.

### Study 2

In this study, we wanted to replicate the results of Study 1 but focused only on high goal importance condition, as only in this condition task rules played an important role. We also wanted to check whether the relationship between NFC and multitasking performance holds when cognitive ability is controlled for. Therefore, we controlled for participants’ working memory capacity, a predictor of performance on many cognitive tasks, especially those that require task switching and multitasking (e.g., Bühner et al. 2006; Colom et al. 2010; Hambrick et al. 2010; König et al. 2005).

#### Method

##### Participants

Ninety-one students^2^ (69 women, 22 men) aged between 18 and 50 (*M* = 22.53, *SD* = 3.77) took part in the study. They were recruited by an announcement and given a monetary compensation of 2.5 EUR in exchange for participation in the study. The study was conducted in line with the local Faculty Ethics Committee with written informed consent from all subjects.

Outlier diagnostics indicated one unusual case (Cook’s distance was equal to 0.11 and studentized deleted residual was equal to 3.94 which were largely too high based on criteria in Fox, 1991). It turned out that this subject was the oldest participant (50 years of age). It was thus deleted from further analyses. Final sample comprised *N* = 90 subjects (68 women, 22 men) with the mean age of *M* = 22.22 (*SD* = 2.41).

##### Measures

The same Need for Cognitive Closure Scale was used as in Study 1 (Cronbach’s α = 0.77; *M* = 3.89, *SD* = 0.67). Participants were also presented with the same dual-tasking procedure. As in Study 1, we manipulated task rules and asked participants to perform both presented tasks as best as possible (dual-tasking condition) or focus on the searching task only (mono-tasking condition). There was no goal importance manipulation (all participants were told that the task measured an important ability which was useful in everyday life and predictive of future academic and job outcomes). After performing a task, participants were asked to answer the same two items measuring the strategy they adopted during task execution, which served as a measure of compliance with task rules.

To measure working memory capacity, we used the n-back task (Jaeggi et al. 2010). During the task participants monitored a series of letters and indicated whether the current letter matched the one presented n-trials back. There were three difficulty levels: 2-back, 3-back and 4-back with three blocks per each level and 23 trials per block. The results were analyzed with the use of signal detection approach (Donaldson 1992). Correct recognitions were counted as hits, and incorrect recognitions as false alarms. Then, the A′ statistic was calculated. Higher A′ scores indicate better discrimination between correct and incorrect stimuli, i.e. greater working memory capacity.

##### Design and procedure

Participants were asked to fill in an online survey (Need for Cognitive Closure Scale and demographic variables) before coming to the laboratory. In the laboratory, they were asked to complete a dual-tasking procedure in either mono-tasking or dual-tasking condition. After completing the task and answering the two strategy items, participants performed the n-back task. Then they were debriefed and thanked. All computer tasks were displayed on 19 inches LCD monitors. The dual-task was programmed in JavaScript. The n-back task was programmed and run with Inquisit 4.0.

#### Results and discussion

**Fig. 4:**
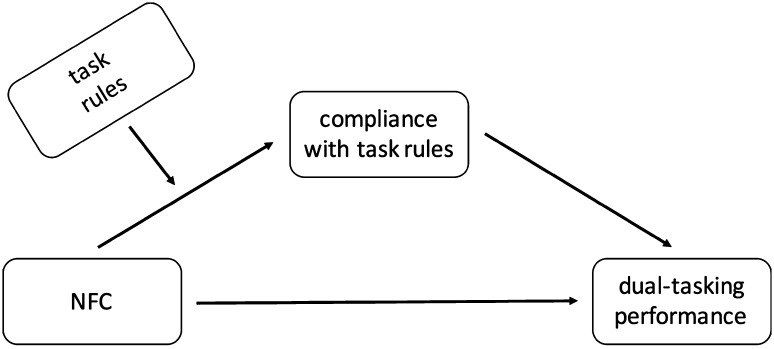
Theoretical model tested in Studies 2 and 3

To test the hypothesis, we regressed the strategy score (a measure of compliance with the task rules) on NFC, condition (task rules), and the interaction between the two variables. We expected that the strategy used in the task would be significantly predicted by the interaction term in a way that high NFC would lead to more priority given to the searching task (i.e., negative effect) in the mono-tasking condition and more priority given to the verification task (i.e. positive effect) in the dual-tasking condition. We then checked whether the strategy score mediated the relationship between NFC and performance depending on the task rules. We thus tested a moderated mediation model as graphically presented in Fig. 4. As in Study 1, we ran separate analyses for measures of mono-tasking and dual-tasking performance.

Moderated mediation analyses were run with the use of the Process macro for SPSS version 2.13, model 7 (Hayes 2013). In all analyses 10,000 bootstrap samples for bias corrected bootstrap confidence intervals were used. Condition was coded as 0 for mono-tasking and 1 for dual-tasking instructions. All continuous variables were standardized before the analyses. Working memory capacity was controlled for.

#### Effects on strategy

**Fig. 5:**
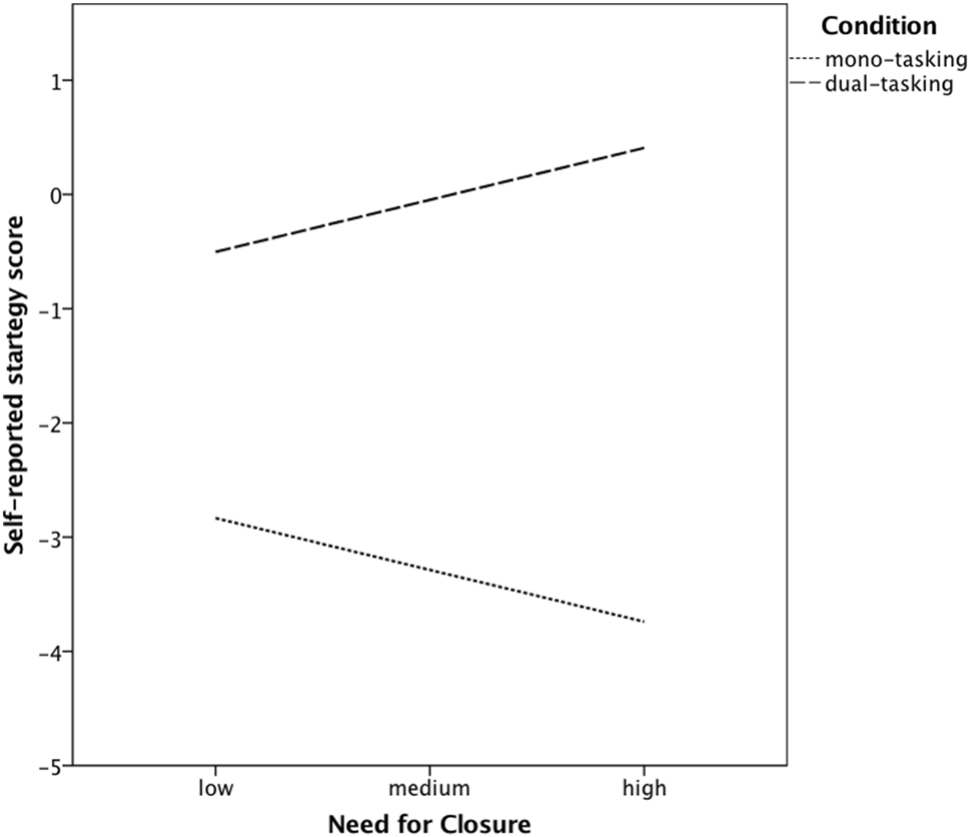
Relationship between need for closure and priority given to the searching vs. verification task, as a measure of compliance with task rules, in both, mono-tasking and dual-tasking, conditions (Study 2)

As presented in Table 2, there was a significant positive effect of the task rule manipulation on strategy. More importantly, however, the effect was qualified by a significant NFC × task rules interaction. As expected, in the mono-tasking condition there was a negative relationship between NFC and strategy (i.e., NFC was related to greater prioritization towards the searching task), whereas in the dual-tasking condition the relationship was positive (i.e., NFC was related to a greater focus on the verification task compared to the mono-tasking condition resulting in more balanced distribution of task focus). The effects were equal to *β* = − 0.20, SE = 0.10, *t* = − 2.04, *p* = .04, 95% CI [− 0.40, − 0.01] and *β* = 0.21, SE = 0.10, *t* = 2.06, *p* = .04, 95% CI [0.01, 0.40] for mono-tasking and dual-tasking conditions, respectively. The interaction is graphically presented in Fig. 5. Additional inspection of differences between conditions showed that they were significant for both, low and high NFC individuals (in both cases *p* < .001).

**Table 2:** Standardized regression coefficient for the model predicting strategy in Study 2 (*N* = 90)

	*β*	SE	*t*	*p*
Step 1^a^				
NFC	0.02	0.08	0.24	.808
Task rules	1.47	0.15	10.01	< .001
Working memory capacity	0.08	0.08	1.03	.308
Step 2^b^				
NFC	− 0.20	0.10	− 1.98	.051
Task rules	1.47	0.14	10.54	< .001
NFC × task rules	0.44	0.14	3.11	.003
Working memory capacity	0.11	0.07	1.52	.133

^a^R^2^ = .54

^b^R^2^ = .58, ∆R^2^ = 0.05, *F* (1, 85) = 9.69, *p* = .003

#### Moderated mediation effects on performance

We then checked whether strategy significantly predicted mono-tasking and dual-tasking performance and tested the overall moderated mediation model. And so, mono-tasking performance was significantly predicted by strategy, *β* = − 0.58, SE = 0.08, *t* = − 6.92, *p* < .001. Also, there was a hypothesized moderated mediation effect (index of moderated mediation, *IMM* = − 0.26, SE = 0.08, 95% CI [− 0.44, − 0.11]). The indirect effect of NFC on mono-tasking performance through strategy was equal to *β* = 0.11, SE = 0.06, 95% CI [0.003, 0.25] in the mono-tasking condition and *β* = − 0.14, SE = 0.05, 95% CI [− 0.27, − 0.05] in the dual-tasking condition. Thus, the higher NFC, the better mono-tasking performance but only in condition in which participants were instructed to give priority to a target task. On the contrary, in condition in which participants were instructed to divide their attention between the two tasks, high NFC was, indirectly through strategy used during task execution, related to poorer accuracy in the searching tasks. The direct effect of NFC was not significant, *β* = − 0.05, SE = 0.09, *t* = − 0.56, *p* = .57, 95% CI [− 0.22, 0.12].

On the other hand, strategy score predicted dual-tasking performance positively, *β* = 0.58, SE = 0.08, *t* = 6.87, *p* < .001. There was also a significant moderated mediation effect (*IMM* = 0.24, SE = 0.08, 95% CI [0.09, 0.42]). As predicted, NFC was negatively related to dual-tasking performance in the mono-tasking condition, *β* = − 0.12, SE = 0.06, 95% CI [− 0.24, − 0.01], and positively in the dual-tasking condition, *β* = 0.12, SE = 0.05, 95% CI [0.03, 0.24]. Both effects were mediated by strategy, or compliance with the task rules. The direct effect of NFC was non-significant, *β* = − 0.004, SE = 0.09, *t* = − 0.05, *p* = .96, 95% CI [− 0.18, 0.17].

A similar effect was found when performance on the verification task only was analyzed. Strategy was a significant predictor, *β* = 0.73, SE = 0.07, *t* = 10.26, *p* < .001, 95% CI [0.59, 0.87]. There was a significant moderated mediation effect (*IMM* = 0.30, SE = 0.10, 95% CI [0.12, 0.50]) indicating a negative indirect effect of NFC on performance on the verification task in the mono-tasking condition, *β* = − 0.15, SE = 0.07, 95% CI [− 0.30, − 0.01], and positive in the dual-tasking condition, *β* = 0.15, SE = 0.06, 95% CI [0.04, 0.28]. The direct effect of NFC was non-significant, *β* = 0.01, SE = 0.07, *t* = 0.07, *p* = .94, 95% CI [− 0.14, 0.15].

In all cases working memory capacity was controlled for, thus suggesting that the obtained effects are not driven by differences in cognitive capacity (analyses with working memory capacity excluded yielded similar results). Working memory capacity significantly predicted performance on the searching task, *β* = 0.22, SE = 0.09, *t* = 2.59, *p* = .01, verification task, *β* = 0.16, SE = 0.07, *t* = 2.13, *p* = .04, as well as dual-tasking performance, *β* = 0.22, SE = 0.09, *t* = 2.52, *p* = .01 (coefficients from moderated mediation models we tested). As presented in Table 2, it did not predict the strategy score. We also tested whether there was an interactive effect of working memory capacity and condition on strategy. The analyses showed that the effect was not significant, *β* = − 0.02, SE = 0.15, *t* = − 0.16, *p* = .87, which suggests that this is motivational rather than cognitive factors that are responsible for compliance with the task rules. In case of performance, working memory capacity significantly predicted performance on individual tasks, as well as overall dual-tasking performance. These effects seem independent from the effect of NFC.

The results of this study showed that NFC was related to greater compliance with task rules, which translated into better dual-tasking performance in the dual-tasking condition and better mono-tasking performance in the mono-tasking condition. This result might seem surprising given the fact that traditionally NFC has been related to rigid processing styles (Kruglanski and Webster 1996). We should note, however, that the results show that NFC might foster multitasking but only in a condition in which multitasking is a rule. When mono-tasking was a rule, NFC was related to a greater focus on one task. The results suggest that contextual factors might determine whether rigid or flexible way of processing will be adopted by high NFC individuals.

### Study 3

The aim of this study was to replicate the results of previous studies with a different dual-task and a more straightforward measure of strategy used in the task. That is, this time we wanted to use an index in which low scores would reflect focusing on one task, whereas high scores straightforwardly indicate focusing on two tasks at a time. To that aim, we used a dual-task in which the two tasks were equally difficult (we used a dual-task consisting of two verification tasks).

#### Participants

Two hundred young adults (136 women, 64 men) aged between 18 and 40 (*M* = 24.00, *SD* = 4.54) took part in the study. They were recruited by an announcement and given a monetary compensation of 2.5 EUR in exchange for participation in the study. The study was conducted in line with the local Faculty Ethics Committee with written informed consent from all subjects.

Seven participants were excluded from further analyses. Six had a very low accuracy in the target task (verification task on the right) in the mono-tasking condition (five participants scored 0 and one correctly verified only 2 mathematical expressions). At the same time, these participants had high accuracy in the verification task on the left (> 40 in all cases), which might suggest that they had misunderstood the assignment and instead of focusing on the task in the right, they focused on the task on the left. There was also one participant who had a very high number of errors (121 in the task on the left and 107 in the task on the right), which might suggest that (s)he did not care for accuracy. Therefore, these cases were excluded from further analyses and data from *N* = 192 participants was analyzed.

#### Measures

**Fig. 6:**
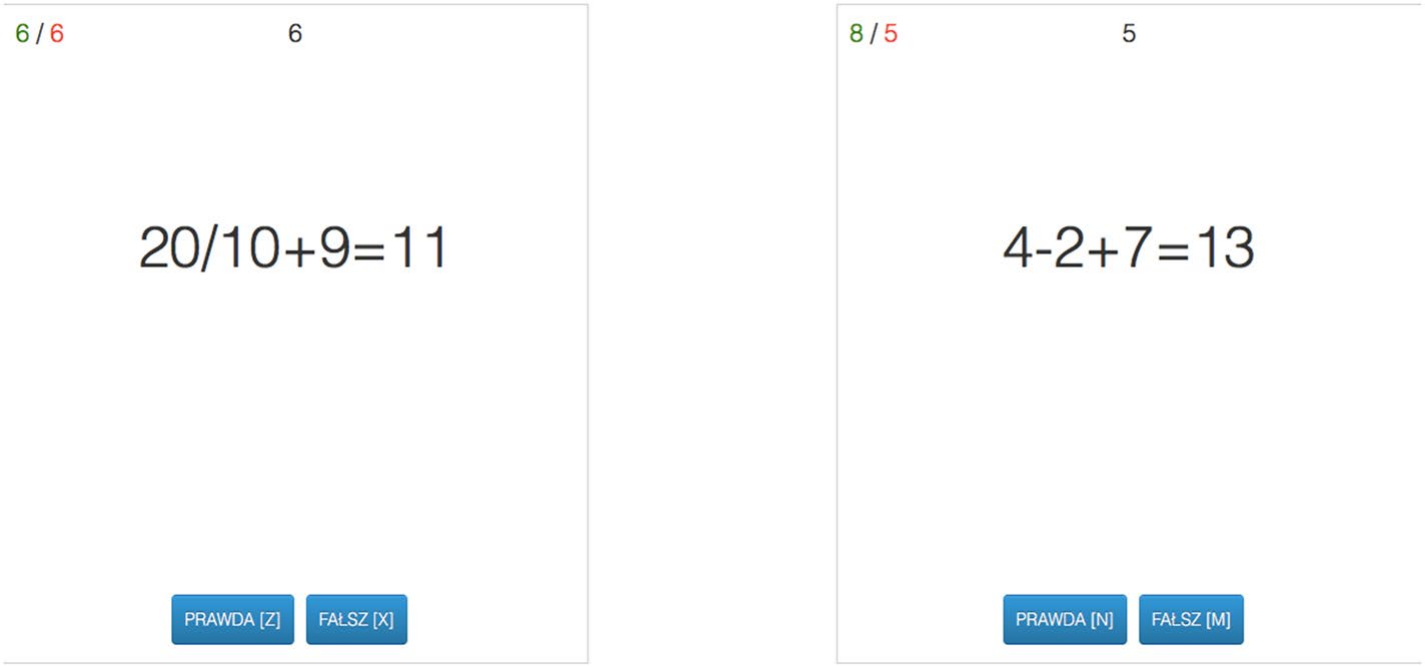
Dual-task procedure used in Study 3. In the dual-tasking condition, participants are asked to perform both tasks as best as possible. In the mono-tasking condition, participants are asked to focus on the task on the right

The same Need for Cognitive Closure Scale was used as in previous studies (Cronbach’s α = 0.85; *M* = 4.03, *SD* = 0.63). To measure dual-tasking performance, we used a modified version of the dual-task we previously used. This time we presented participants with two verification tasks side by side (see Fig. 6). Participants were asked to indicate whether the presented expressions were true or false. For each task, an expression was presented for maximally 7.5 s and changed after that time or when a participant had verified it. Responses were given by pressing one of the response keys on the keyboard: “Z” for true and “X” for false for the task on the left and “N” for true and “M” for false for the task on the right. As a measure of performance we looked at the number of correctly verified expressions in each task. We also computed a multitasking performance index by averaging correct responses to both tasks.

We manipulated task rules in an analogues way as in the previous studies. In the mono-tasking condition, we asked participants to focus only on one task (this time it was the verification task on the right), whereas in the dual-tasking condition we asked participants to focus on the two tasks at the same time.

Strategy was measured with the item: “While performing the task, did you focus on one or two tasks at the same time?” (anchored 0—*entirely on one task*, 10—*on two tasks at the same time*).

#### Design and procedure

The study was run entirely in the lab. Participants were first asked to fill in the NFC scale and then to perform a dual-task procedure in either mono-tasking or dual-tasking condition. After performing the task, participants answered the question about the strategy they had used. After this part, they were also presented with some other questionnaires being a part of a different study. Then, they were debriefed and thanked. The dual-task was programmed in JavaScript and was displayed on 19 inches LCD monitors.

#### Results and discussion

To test our hypotheses, we ran similar analyses as in Study 2 and regressed the strategy score on NFC, condition (task rules), and the interaction between the two variables. We expected that high NFC would be related to more focus on one task (i.e., negative effect on the dual-strategy measure) in the mono-tasking condition and more focus on two tasks (i.e. positive effect on the dual-strategy measure) in the dual-tasking condition. We then checked whether the strategy score mediated the relationship between NFC and performance depending on the task rules (moderated mediation model as graphically presented in Fig. 4).

#### Effects on strategy

**Fig. 7:**
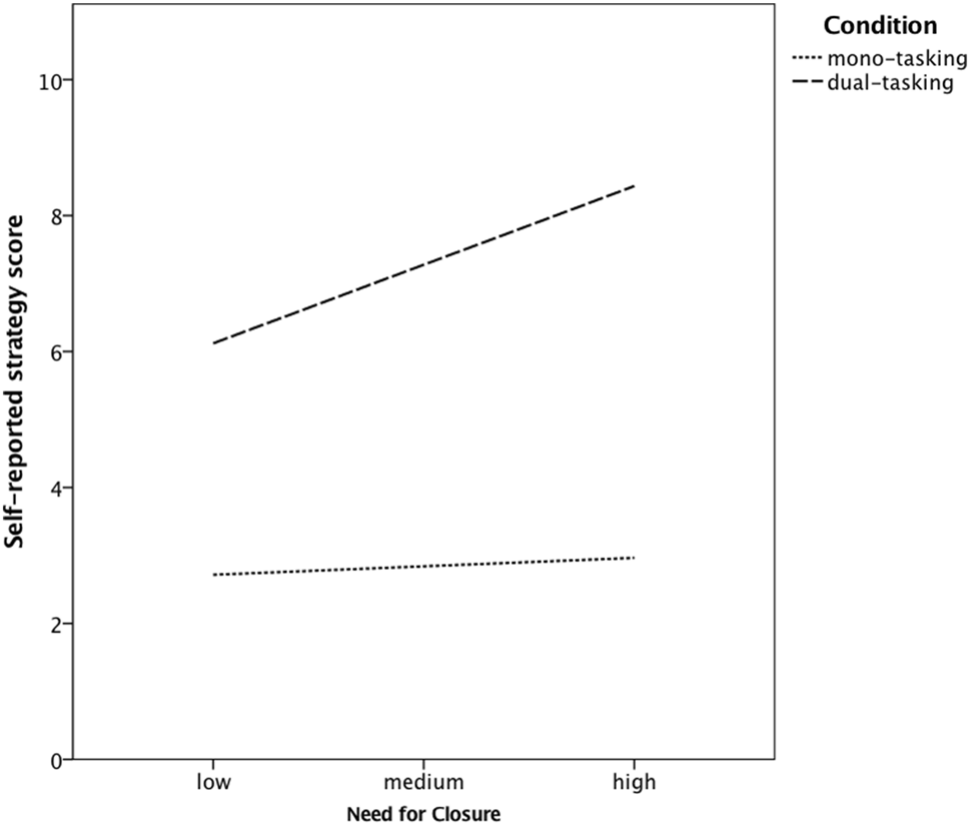
Relationship between need for closure and strategy used in the dual-task in both, mono-tasking and dual-tasking, conditions (Study 3)

As presented in Table 3, there was a significant positive effect of the task rule manipulation on strategy suggesting that in the dual-tasking condition participants focused more on two tasks than in the mono-tasking condition. As predicted, however, this effect was qualified by a significant NFC × task rules interaction (see Table 3). The results indicate that there was a significant effect of NFC on strategy in the dual-tasking condition, *β* = 0.30, SE = 0.10, *t* = 3.13, *p* = .002, 95% CI [0.11, − 0.49], suggesting that the higher NFC, the more dual-tasking strategy when dual-tasking was required. However, the effect in the mono-tasking condition was non-significant, *β* = 0.03, SE = 0.07, *t* = − 0.43, *p* = .67, 95% CI [− 0.12, 0.18]. Inspection of differences between conditions showed that they were significant for both low and high NFC individuals (in both cases p < .001). The interaction is graphically presented in Fig. 7.

**Table 3:** Standardized regression coefficient for the model predicting strategy in Study 3 (N = 192)

	*β*	SE	*t*	*p*
Step 1^a^				
NFC	0.13	0.06	2.25	.026
Task rules	1.14	0.12	9.56	< .001
Step 2^b^				
NFC	0.03	0.08	0.43	.668
Task rules	1.15	0.12	9.71	< .001
NFC × task rules	0.27	0.12	2.20	.029

^a^R^2^ = .33

^b^R^2^ = .35, ∆R^2^ = 0.02, *F* (1, 189) = 4.84, *p* = .029

#### Moderated mediation effects on performance

We then checked whether strategy significantly predicted mono-tasking and dual-tasking performance, and subsequently tested the overall moderated mediation model. Mono-tasking performance (accuracy in the verification task on the right) was significantly predicted by strategy, *β* = − 0.37, SE = 0.07, *t* = − 5.39, *p* < .001. Also, there was a hypothesized moderated mediation effect (*IMM* = − 0.10, SE = 0.05, 95% CI [− 0.22, − 0.01]). The indirect effect of NFC on mono-tasking performance through strategy was significant and negative in the dual-tasking condition, *β* = − 0.11, SE = 0.05, 95% CI [− 0.22, − 0.03], but non-significant in the mono-tasking condition, *β* = − 0.01, SE = 0.02, 95% CI [− 0.06, 0.03]. The direct effect of NFC was not significant, *β* = 0.07, SE = 0.07, *t* = 1.04, *p* = .30, 95% CI [− 0.06, 0.20].

Dual-tasking performance was also significantly predicted by strategy, *β* = 0.26, SE = 0.07, *t* = 3.63, *p* < .001. There was also a significant moderated mediation effect (*IMM* = 0.07, SE = 0.04, 95% CI [0.01, 0.15]). As predicted, NFC was indirectly positively related to dual-tasking performance in the dual-tasking condition, *β* = 0.08, SE = 0.03, 95% CI [0.02, 0.16], so the higher NFC, the better dual-tasking performance in the dual-tasking condition. The effect in the mono-tasking condition was not significant, *β* = 0.01, SE = 0.02, 95% CI [− 0.02, 0.04]. The direct effect of NFC was non-significant, *β* = 0.02, SE = 0.07, *t* = 0.22, *p* = .83, 95% CI [− 0.12, 0.15].

We also analyzed performance for the verification task on the left (the task to be ignored in the mono-tasking condition). And, again, strategy was a significant predictor, *β* = 0.64, SE = 0.06, *t* = 11.27, *p* < .001, 95% CI [0.52, 0.75], so the more dual-tasking strategy participants adopted, the better performance on this task. There was also a significant moderated mediation effect (*IMM* = 0.17, SE = 0.08, 95% CI [0.01, 0.32]). The effect of NFC was significant in the dual-tasking, *β* = 0.19, SE = 0.07, 95% CI [0.05, 0.33], but not in the mono-tasking condition, *β* = 0.02, SE = 0.04, 95% CI [− 0.05, 0.09]. The direct effect of NFC was non-significant, *β* = − 0.05, SE = 0.06, *t* = − 0.83, *p* = .41, 95% CI [− 0.16, 0.06].

The results of the study replicated the findings of previous studies indicating the interactive effect of NFC and task rules on strategy, and indirectly—on performance. Like in previous studies, NFC was related to better dual-tasking performance but only in the dual-tasking condition (indirect effect via strategy). This provides further support for the notion that high NFC individuals can be better multitaskers in certain situations. In this study, however, we did not find effects in the mono-tasking condition. As presented in Fig. 7, all participants tended to focus on one task to the same extent, i.e., regardless of their NFC level. The lack of a significant effect in this condition (with significant effects in previous studies) might stem from the fact that a different task to be focused on was used. In previous studies, the target task in the mono-tasking condition was easier, thus leaving more free resources to be engaged in another activity. Also, refreshing rate of the searching task in the previous studies was fixed and did not depend on participants’ activity, whereas in the verification task participants were presented with a new expression to be verified once they provided a response to the previous one. Therefore, participants could adjust the presentation tempo to their preferences and get fully occupied with this sole task. In case of the searching task, participants had to wait for a new letter to appear, therefore focusing only on this task required more focus and inhibitory control in order to resist the temptation to engage in another activity (which might be especially tempting for low NFC individuals who prefer multitasking over focusing on one task at a time, Szumowska et al. 2017).

## General discussion

The aim of the studies presented in this paper was to take a closer look at the role that contextual factors, such as task rules, play in multitasking performance. We postulated that task rules should be especially important for high NFC individuals, who are highly motivated to adhere to them. Task rules should thus drive (moderate) the behavior of high NFC individuals to a greater extent than behavior of low NFC individuals. So, higher NFC levels should be related to a more multitasking strategy when multitasking is imposed by the task rules and more mono-tasking strategy when monotasking is imposed by the task rules. We further expected that this grater rule compliance should translate into respective differences in performance. We expected this effect to be significant when goal importance was high, but not when it was low.

The results were supportive as it turned out that NFC was related to more priority given to a target task in the mono-tasking condition (Studies 1 and 2) and to more dual-tasking strategy in the dual-tasking condition (Studies 1–3). This translated into differences in performance, as NFC was positively related to mono-tasking performance and negatively to dual-tasking performance in the mono-tasking condition. By contrast, it was positively related to dual-tasking performance and negatively to mono-tasking performance in the dual-tasking condition. All effects were mediated by compliance with the task rules and were significant only when goal importance was high, but not when it was low. This suggests that the effects we have obtained can be accounted for by differences in motivation.

The results are thus in line with theories emphasizing the role of goal importance in determining the magnitude of motivation (e.g., Atkinson and Birch 1970; Lewin et al. 1944; Vroom 1964). They are also in accordance with the Cognitive Energetics Theory (Kruglanski et al. 2012) or Motivational Intensity Theory (Brehm and Self 1989) in the sense that they show that high goal importance is necessary for individuals to invest effort. However, how much resources are invested in a given activity directly depends on the task demands—in our studies NFC was related to a multitasking (more effortful) strategy only when it was required. When mono-tasking (less effortful) strategy was required, high NFC was related to a greater focus on one task only. So, when the goal to comply with the task demands could be satisfied with the use of a less effortful strategy, the difficult one was not adopted (similar was found by Sankaran et al. 2017, and; Szumowska et al. 2017; see also; Richter et al. 2012; Wright 1996, 2008).

The results we have obtained add also to the NFC theory. They show that NFC does not always lead to effort minimizing strategy of task performance. Rather, they suggest that which strategy would be used depends on contextual factors, or task rules. This is in line with other results showing that NFC might lead to effortful processing in certain circumstances (Jaśko et al. 2015; Kruglanski et al. 1991, 1993). The results also corroborate previous findings showing that high NFC participants are more motivated to comply with task demands, even if such behavior requires more effort (Jaśko et al. 2015; Jia et al. 2014). They additionally show that contextual factors play a moderating role in the relationship between NFC and behavior, which is in accordance with results obtained by Chiu et al. (2000) or Fu et al. (2007). Also, these findings provide further evidence that whereas behaviour of high NFC individuals strongly depends on the context, it is not so much the case for low NFC individuals who are less sensitive to situational and normative pressures and less prone to adjust to them (e.g., Jaśko et al. 2015; Kossowska et al. 2016; Kruglanski et al. 2006).

Our studies show that NFC is related to greater adherence to task rules, and hence better mono- or multitasking performance, depending on these rules. However, it seems plausible that other contextual cues might play a similar role. Previous studies show that high NFC individuals conform to cultural and situational norms to a greater extent (Chiu et al. 2000; Fu et al. 2007). They might thus adjust to organizational culture or climate to a greater extent and multitask more in organizations that promote multitasking (polychronic ones) and work on one task at a time in organizations that promote the same (monochronic ones, Bluedorn et al. 1992; Slocombe and Bluedorn 1999). This might be especially relevant in today’s corporate environment which is characterized by high levels of complexity, uncertainty, and unpredictability (Benabou 1999; Delbridge 2000). Dealing with uncertainty is especially important for high NFC individuals. Therefore, the role of contextual cues in guiding their behavior in such environments might be even greater.

Our results have also important implications for individual and organizational performance. Not only do they support the finding that NFC is a significant predictor of multitasking performance, but also show that contextual factors and their interaction with NFC play an important role. Although multitasking might be more difficult for high NFC individuals and they perform worse on such tasks when NFC-related deficiencies cannot be compensated for (Szumowska and Kossowska 2016), they might exhibit better multitasking performance due to their greater motivation to comply with task rules. That is, motivational effort might compensate for these deficiencies or even lead to better outcomes. However, there are two important issues that need to be noted here. The first relates to a more general question of the relationship between motivation (effort) and performance. In our study greater motivation to comply with task rules led to better performance. However, it is not always the case. To the contrary, it has been argued that the relationship between effort and performance is complex and not straightforward (Silvia et al. 2013). So, the results we have obtained should not be generalized to all tasks. The procedure we have used relies more on task switches rather that division of attention, thus with the use of effortful control, the same or better performance might be obtained even for individuals with smaller pool of resources (i.e., individuals with high NFC, Kossowska 2007a, b; Kossowska et al. 2010). But in tasks that rely more on a fixed pool of attentional resources (typical dual-tasking and divided attention paradigms, see Pashler 1994, for examples), effort would not compensate for limited resource pool. When there are less resources to be shared, and task performance depends on the amount of resources allocated to each task at the same time (rather than shifting attentional resources from one task to another), different effects could be found. Similar should be the case when more frequent switches between tasks were required or tasks were generally more difficult. This is in line with previous studies showing that high NFC was related to better performance on a task when it was accompanied by another task but only when the task was easy and participants’ shifting ability was high. When the difficulty level was higher, no positive effect of NFC was found (Szumowska and Kossowska 2016).

Secondly, if better dual-tasking performance of high NFC individuals in the dual-tasking condition stems from more effort exerted to perform this task (high NFC individuals try harder in order to comply with task rules), they might experience fatigue sooner than individuals low on NFC when required to multitask for a long time. It can also affect their level of stress, coping and withdrawal (Delbridge 2000). The threshold above which the tasks are unmanageable might also be lower for high than low NFC individuals. Moreover, it can have consequences for well-being, as previous studies show that a misfit between a person’s preference for multitasking and multitasking requirements is related to lower job satisfaction, lower self-efficacy and higher psychological strain (Hecht and Allen 2005). Thus, even though high NFC individuals might be effective multitaskers and outperform their low NFC counterparts under some circumstances, there might be some long-distance costs such as lower well-being and satisfaction. Also, studies by Slocombe and Bluedorn (1999) show that congruency between person’s preferred and perceived work-unit’s level of multitasking is related to organizational commitment, perceived fairness of evaluation and perceived level of performance. Thus, incongruency in that matter (high NFC individuals in a highly multitasking environment) might lead to the opposite effects. This, however, calls for further research, preferably in a natural organizational setting.

We should also note that in this paper we hypothesized (Figs. 2, 4) and tested indirect (not direct) effects of NFC on performance (also, we did not treat the presence of a direct or total effect as a prerequisite to searching for evidence of indirect effects, Hayes 2013; LeBreton et al. 2009; MacKinnon 2008; Rucker et al. 2011; Shrout and Bolger 2002; Zhao et al. 2010). We expected NFC to be related to a greater compliance with the task rules, which should translate into better mono-tasking or dual-tasking performance, depending on a condition. The results were supportive. This, however, is different from testing the interactive effect of NFC and task rules directly on performance. We did not hypothesize a direct effect of on performance as (multitasking) performance depends not only on one’s motivation but also on their ability (e.g., Bühner et al. 2006; Colom et al. 2010; Hambrick et al. 2010; König et al. 2005). In line with that, previous studies show that there is no direct link between NFC and multitasking performance, unless moderating factors are included (e.g., shifting ability, Szumowska and Kossowska 2016). Here, we focused on the motivational effect only (and as shown by Study 2, this effect is independent from the influence of cognitive factors, such as working memory capacity). Including such factors, however, would be a good direction of future studies, as it would be valuable to see how ability factors moderate the efficiency of strategy implementation.

## Footnotes

1 A priori power analysis (*G***Power* 3.1, Faul et al. 2009) for a regression model testing the interaction effects with a medium effect size (*f*^2^ = .15) showed that a sample size of at 85 would be sufficient to obtain a statistical power at the recommended level of .80 (Cohen 1988). But since we tested a moderated mediation hypotheses also, we recruited more participants.

2 A priori power analysis (*G***Power* 3.1, Faul et al. 2009) for a regression model testing the interaction effect with an effect size of *f*^2^ = .09 (based on the results of Study 1 for the NFC × task rules interaction in the high goal importance condition) showed that a sample size of at least 90 would be sufficient to obtain a statistical power at the recommended level of .80 (Cohen 1988).
